# Mitochondrial fission and mitophagy are independent mechanisms regulating ischemia/reperfusion injury in primary neurons

**DOI:** 10.1038/s41419-021-03752-2

**Published:** 2021-05-12

**Authors:** Anthony R. Anzell, Garrett M. Fogo, Zoya Gurm, Sarita Raghunayakula, Joseph M. Wider, Kathleen J. Maheras, Katlynn J. Emaus, Timothy D. Bryson, Madison Wang, Robert W. Neumar, Karin Przyklenk, Thomas H. Sanderson

**Affiliations:** 1 Department of Emergency Medicine, University of Michigan Medical School, Ann Arbor, MI 48109 USA; 2 Department of Physiology, Wayne State University School of Medicine, Detroit, MI 48201 USA; 3 Department of Human Genetics, University of Pittsburgh, Pittsburgh, PA 15269 USA; 4 Neuroscience Graduate Program, University of Michigan Medical School, Ann Arbor, MI 48109 USA; 5 Frankel Cardiovascular Center, University of Michigan Medical School, Ann Arbor, MI 48109 USA; 6 Department of Molecular and Integrative Physiology, University of Michigan Medical School, Ann Arbor, MI 48109 USA

**Keywords:** Mitophagy, Mechanisms of disease

## Abstract

Mitochondrial dynamics and mitophagy are constitutive and complex systems that ensure a healthy mitochondrial network through the segregation and subsequent degradation of damaged mitochondria. Disruption of these systems can lead to mitochondrial dysfunction and has been established as a central mechanism of ischemia/reperfusion (I/R) injury. Emerging evidence suggests that mitochondrial dynamics and mitophagy are integrated systems; however, the role of this relationship in the context of I/R injury remains unclear. To investigate this concept, we utilized primary cortical neurons isolated from the novel dual-reporter mitochondrial quality control knockin mice (C57BL/6-Gt(ROSA)26Sortm1(CAG-mCherry/GFP)Ganl/J) with conditional knockout (KO) of *Drp1* to investigate changes in mitochondrial dynamics and mitophagic flux during in vitro I/R injury. Mitochondrial dynamics was quantitatively measured in an unbiased manner using a machine learning mitochondrial morphology classification system, which consisted of four different classifications: network, unbranched, swollen, and punctate. Evaluation of mitochondrial morphology and mitophagic flux in primary neurons exposed to oxygen-glucose deprivation (OGD) and reoxygenation (OGD/R) revealed extensive mitochondrial fragmentation and swelling, together with a significant upregulation in mitophagic flux. Furthermore, the primary morphology of mitochondria undergoing mitophagy was classified as punctate. Colocalization using immunofluorescence as well as western blot analysis revealed that the PINK1/Parkin pathway of mitophagy was activated following OGD/R. Conditional KO of *Drp1* prevented mitochondrial fragmentation and swelling following OGD/R but did not alter mitophagic flux. These data provide novel evidence that Drp1 plays a causal role in the progression of I/R injury, but mitophagy does not require Drp1-mediated mitochondrial fission.

## Introduction

Mitochondrial quality control (MitoQC), consisting of mitochondrial dynamics and mitophagy, has been a major area of focus in the context of many disease conditions including cerebral ischemia/reperfusion (I/R) injury. Mitochondrial damage and subsequent dysfunction have been well characterized as precursors to cell death following I/R^1–4^. Therefore, it is critical to have stringent quality control mechanisms in place to sequester and dispose of damaged mitochondria.

Multiple stressors (including cerebral I/R) have been demonstrated to initiate a transition to a fragmented mitochondrial phenotype, and there is a consensus that stress-induced activation of mitochondrial fission is directly linked to cell death^5–9^. Moreover, these stressors, and the accompanying increase in mitochondrial fission, are also reportedly associated with an upregulation in mitophagy^10,11^. However, despite the importance of these insights, two major gaps in knowledge remain. First, it is unknown whether fission is necessary for the upregulation of mitophagy and subsequent disposal of damaged mitochondria following I/R injury. Second, there is no agreement on whether the upregulation of mitophagy observed in the setting of I/R is beneficial or detrimental to neuronal viability^12–17^. Indeed, past studies underscore a complex and seemingly disparate relationship between mitochondrial dynamics and mitophagy, i.e., inhibiting fission during ischemia may attenuate cell death^8,13,18,19^, or, conversely, may worsen outcomes by compromising the clearance of damaged mitochondria that accumulate during reperfusion. To further complicate the roles and relationships between fission and dynamics in the context of I/R, mitophagy is a dynamic process, and it has been technically challenging to reliably quantify overall mitophagic flux in an unbiased manner^12–17^.

Dynamin-related protein 1 (Drp1) is considered the “master regulator” of mitochondrial fission. However, the role of Drp1 may extend beyond the regulation of fission per se; Drp1 has also been implicated as a component of the mitophagic process, responsible for sequestering damaged portions of the mitochondrial network for recycling through mitophagy/biogenesis^10,20,21^. For example, previous studies have demonstrated that overexpression of Drp1 in HeLa cells resulted in a 70% decrease in mitochondrial mass^10^, suggesting a direct connection between induction of fission, activation of mitophagy, and degradation of mitochondria. Further supporting this connection between fission and mitophagy, Endophilin B1, a Drp1-dependent mediator of fission, was found to colocalize with autophagic markers LC3, Atg5, and Atg9 in response to nutrient starvation^10,20^. Finally, overexpression of Parkin in N2a neuroblastoma cells purportedly protects against I/R injury through ubiquitination and proteasomal degradation of Drp1^21^. These connections suggest a potential mechanism where Drp1-dependent fission would aid in recovery from I/R injury by: (i) inducing fragmentation of damaged/dysfunctional mitochondrial segments, and (ii) facilitating mitophagic degradation.

To provide a robust test of this as-yet unproven concept, we utilized MitoQC reporter mice (C57BL/6-Gt(ROSA)26Sortm1(CAG-mCherry/GFP)Ganl/J)^22^, with and without conditional knockout (KO) of *Drp1*, to quantify changes in: (i) mitochondrial dynamics, and (ii) mitophagic flux (assessed using a novel and unbiased machine learning algorithm) in an in vitro model of cerebral I/R injury. Specifically, we hypothesized that simulated I/R (i.e., oxygen-glucose deprivation (OGD) followed by reoxygenation (OGD/R)) would induce mitochondrial fragmentation and activation of PINK1/Parkin-mediated mitophagy. Moreover, we posited that *Drp1*KO would prevent mitochondrial fragmentation and swelling and limit mitophagy.

## Results

### Mitophagy is upregulated during OGD/reoxygenation

**Fig. 1:**
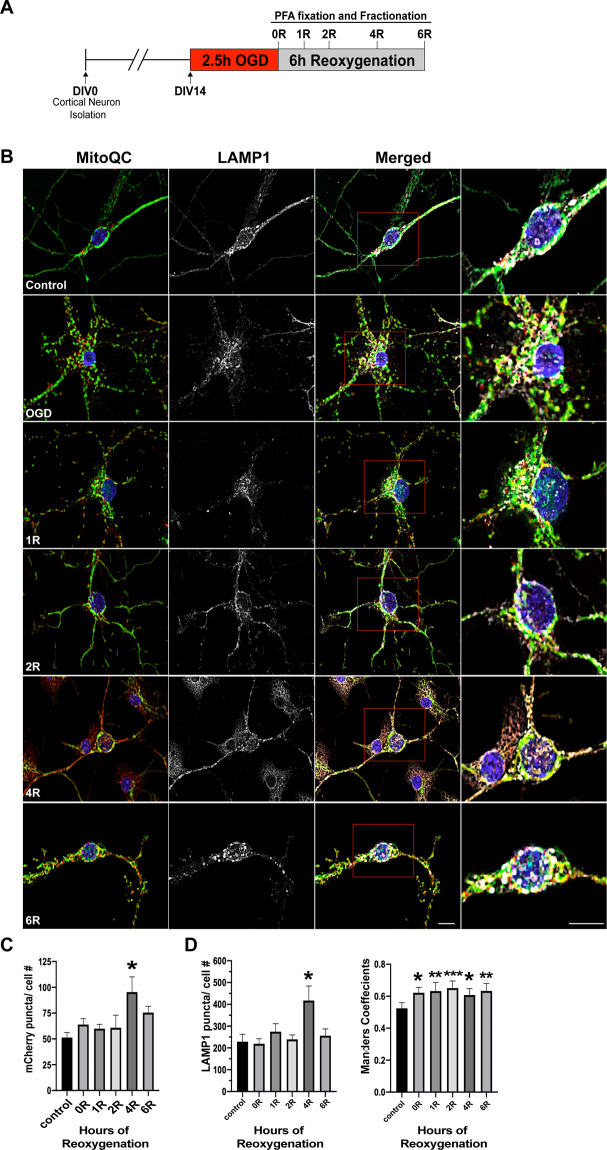
Mitophagic flux is increased following OGD/R. **A** Schematic diagram of experimental design. **B** Utilizing primary cortical neurons from MitoQC reporter mice, cells were analyzed for the presence of mCherry puncta. Cells containing MitoQC were also stained with LAMP1, a lysosomal marker, to determine colocalization of mCherry puncta with lysosomes. **C** Quantification of mCherry puncta normalized to cell count. **D** Mander’s correlation coefficient was used to quantify colocalization of mCherry with LAMP1 throughout OGD/R. LAMP1 particle counts were analyzed and normalized to cell count. Differences across time were analyzed using one-way ANOVA with Tukey post-hoc analysis for multiple comparisons. R post-reoxygenation; *p < 0.05, **p < 0.01; ***p < 0.001 vs controls; *n* = 6 biological replicates. Scale bar = 10 µm.

Previous studies have reported upregulation of mitophagy following cerebral I/R by demonstrating increased expression of key autophagy markers and/or increased colocalization of mitochondria with lysosomes^13–16^. To establish whether mitochondria are undergoing active degradation during I/R, we utilized primary cortical neurons isolated from MitoQC mice^22^. Neurons were subjected to OGD/R and analyzed for the accumulation of mCherry fluorescence in the absence of mitochondrial GFP fluorescence (mCherry puncta) as a marker for mitochondria undergoing mitophagy (Fig. 1A, B). Counts were normalized to cell number to provide average mCherry object counts per cell. Our results show that mCherry puncta were significantly increased at 4 h post R vs control (Fig. 1C). mCherry puncta return to baseline levels at 6 h post R suggesting ongoing mitochondrial degradation by lysosomes. To validate that mCherry puncta were a marker of mitochondria inside lysosomes, we co-labeled MitoQC primary neuron cultures with LAMP1 (a lysosome marker) and analyzed the number of LAMP1 puncta per cell as well as colocalization of mCherry with Lamp1 fluorescence (Fig. 1B). Quantification using Mander’s correlation identified significant increases in colocalization across all time points post R vs control (Fig. 1D). Consistent with changes in mCherry fluorescence, the number of Lamp1 puncta was significantly increased at 4 h post R vs control (Fig. 1D). I/R injury has been associated with increases in autophagy to prevent the accumulation of dysfunctional subcellular components and organelles including mitochondria^13,23^, thus explaining the increase in Lamp1-positive puncta. Consistent with previous studies using MitoQC^22,24,25^, these data validate mCherry puncta as an index of mitophagic flux. Moreover, our results demonstrate that mitophagic flux is significantly increased at 4 h following OGD.

### PINK1/Parkin pathway is activated by OGD/reoxygenation

Mitophagy is activated through multiple mechanisms^26–28^, with the PINK1/Parkin pathway being the most thoroughly investigated. The PINK1/Parkin pathway has been implicated as a major therapeutic target for various neurodegenerative diseases and previous studies have suggested its involvement in I/R injury in both heart and brain^13,16,29–33^. During periods of mitochondrial stress, as in I/R, mitochondrial ROS destabilize the inner mitochondrial membrane through oxidation of protein complexes and mitochondrial lipids^3,4^. Inner mitochondrial membrane destabilization and mitochondrial depolarization allows the accumulation of PINK1 on the mitochondria and subsequent recruitment of Parkin and activation of mitophagy^34–38^.

**Fig. 2:**
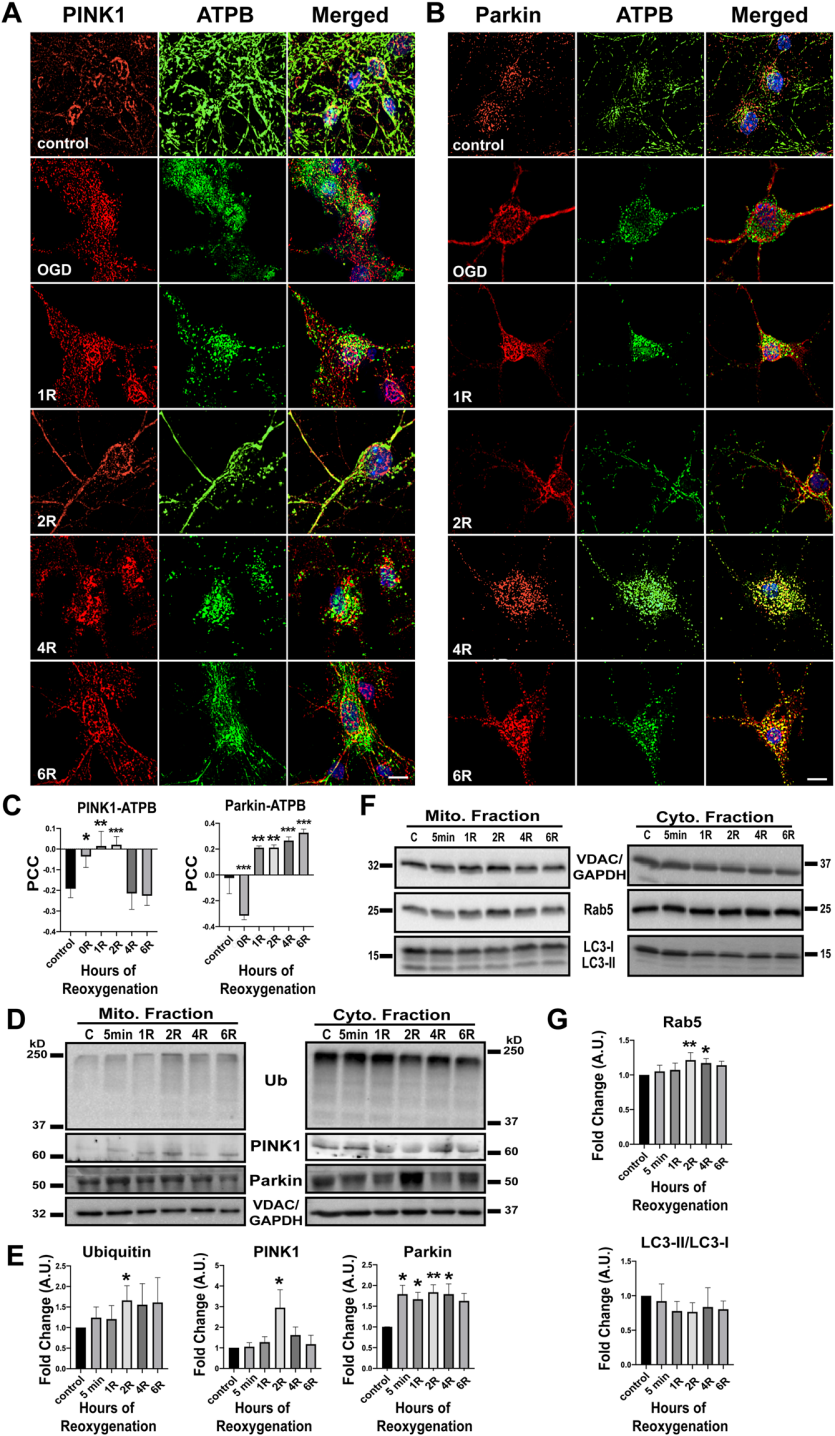
PINK1/Parkin-mediated mitophagy. **A** Cells labeled with PINK1 and ATPB for colocalization of PINK1 with mitochondria, *n* = 4. **B** Cells labeled with Parkin and ATPB for colocalization of Parkin with mitochondria, *n* = 3. **C** Quantification of colocalization using Pearson’s correlation coefficient (PCC). **D** Western Blot of mitochondrial and cytosolic fractions for PINK1, Parkin, and ubiquitin. **E** Quantitation of protein levels, normalized to VDAC and GAPDH, Ubiquitin: *n* = 6, PINK1: *n* = 5, Parkin: *n* = 8. **F** Western Blot of LC3 (autophagy marker) and Rab5 (endosome marker) in mitochondrial and cytosolic fractions. **G** Quantitation of LC3 conversion and Rab5, normalized to VDAC and GAPDH, LC3: *n* = 4, Rab5: *n* = 4. Differences between groups were computed using one-way ANOVA with Dunnett’s post-hoc analysis for comparisons versus control. R post-reoxygenation; *p < 0.05; **p < 0.01; ***p < 0.001. Scale bar = 10 µm.

Accordingly, we examined the role of the PINK1/Parkin pathway in upregulating mitophagy observed in our model of neuronal OGD. To investigate both PINK1 and Parkin translocation to mitochondria following OGD/R, wild-type (WT) neurons were immunolabeled with PINK1 or Parkin (red) and co-labeled for the β-subunit of ATP synthase (ATPB—green) as a marker of mitochondria (Fig. 2A, B). Colocalization was analyzed using Pearson’s correlation coefficient in ImageJ. Analysis of PINK1 fluorescence revealed significant colocalization with ATPB during early reoxygenation vs control, with a peak response observed at 2 h post R (Fig. 2A). Parkin colocalization was significantly decreased during OGD, and significantly increased after 1, 2, 4, and 6 h of reoxygenation, compared to control (Fig. 2B).

In support of the concept that OGD/R-induced mitophagy is mediated through the PINK1/Parkin pathway, western blot analysis revealed an increase of PINK1 and Parkin in mitochondrial fractions (Fig. 2C). The presence of PINK1 in mitochondrial fractions was significantly increased from control at 2 h post R (Fig. 2D), matching the increases in colocalization of PINK1 with ATPB (Fig. 2A). The presence of Parkin in mitochondrial fractions was significantly increased throughout reoxygenation vs control (Fig. 2D), consistent with the outcomes obtained with colocalization analysis (Fig. 2B).

Taken together these data suggest that PINK1 translocation, which occurs early in reoxygenation and peaks at 2 h post R, precedes activation of mitophagy, as indicated by MitoQC reporter data. In addition, these results suggest that Parkin translocation occurs during early reoxygenation and persists through late reoxygenation, perhaps until dysfunctional mitochondria are cleared. This latter concept is supported by the findings of Ordureau et al., reporting evidence of a feed-forward mechanism of Parkin translocation and mitochondrial ubiquitination^39^. To investigate this possibility, Parkin activity was analyzed by ubiquitination of mitochondrial proteins via western blot (Fig. 2C). Mitochondrial ubiquitination was significantly increased at 2 h post R when compared with control, corresponding to the peak increases observed in PINK1 and Parkin translocation (Fig. 2D).

### Endosomal Rab5 expression is increased in mitochondrial fractions

Mitochondria tagged for degradation are cleared through two distinct pathways: autophagosomal-mediated degradation and Rab5 endosomal-mediated degradation. Each pathway involves: (i) identification of dysfunctional mitochondria, (ii) sequestration through either the phagophore or early endosomes, respectively, and (iii) degradation of mitochondria via fusion of the autophagosome or late endosome with the lysosome^40–42^. To characterize the pathway of mitochondrial clearance activated in this model, we probed mitochondrial fractions via western blot for LC3 conversion via proteolytic activation of LC3-I to LC3-II as an indicator of autophagosomal-mediated degradation, and for the presence of Rab5 as an indicator of endosomal-mediated degradation (Fig. 2E). Following OGD, expression of the endosomal protein Rab5 was increased in mitochondrial fractions at 2 and 4 h post R vs control (Fig. 2F), while conversion of LC3-I to LC3-II remained unchanged. These data suggest that mitochondria tagged for degradation by the PINK1/Parkin pathway are being sequestered by Rab5 endosomes for transport to the lysosome following OGD/R, and not through pathways involving LC3 conversion.

### Mitochondrial morphology classification—machine learning model

**Fig. 3:**
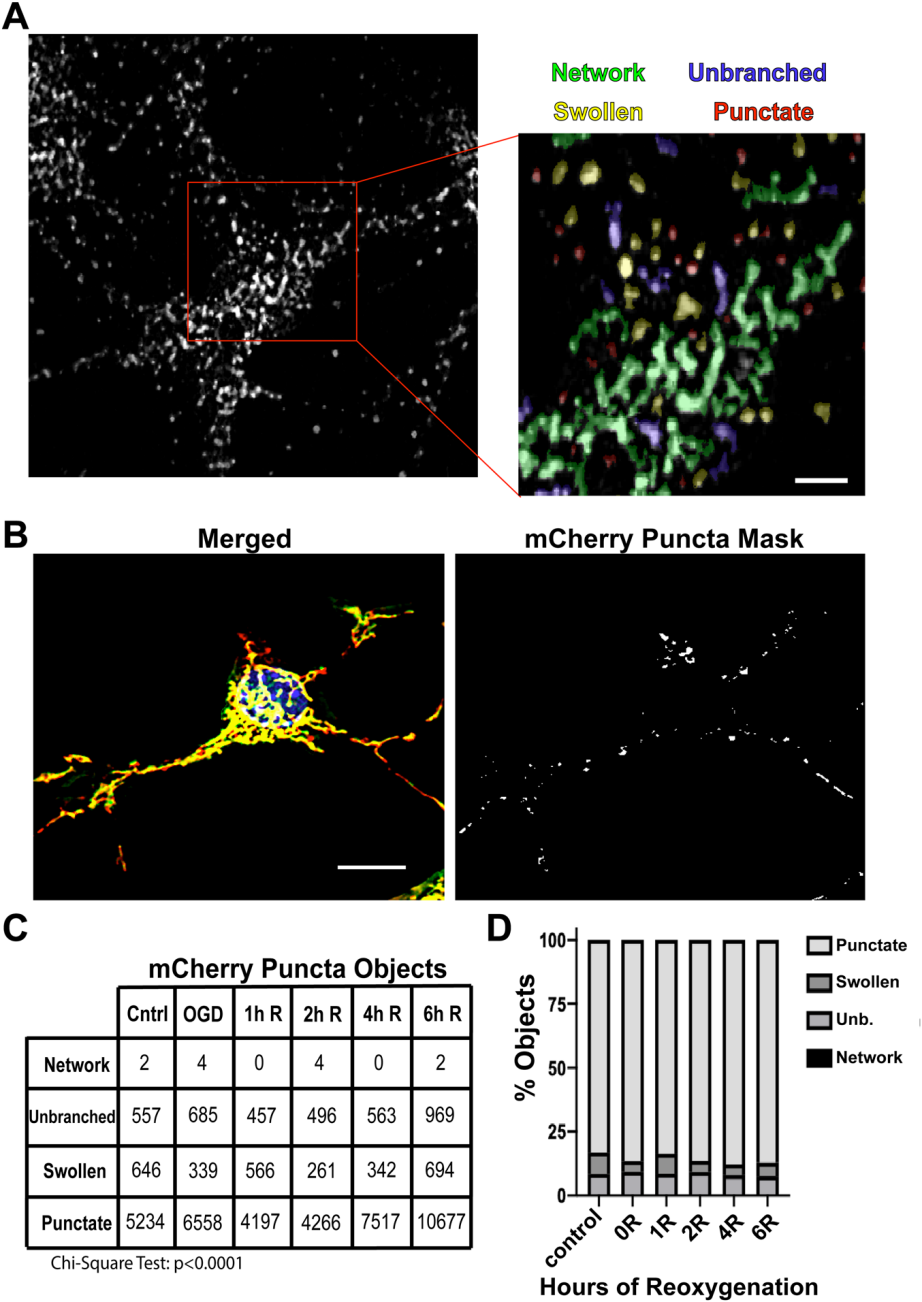
Mitochondria morphology classification of mCherry puncta. **A** Mitochondrial morphology was classified using a machine learning-based classification system. Mitochondria were classified into four different morphologies consisting of: networks (green), unbranched (blue), swollen (yellow), and punctate (red). Scale bar = 5 µm. **B** mCherry puncta were segmented from images of MitoQC neurons and analyzed using our mitochondrial morphology classification system. **C** Total mCherry puncta counts throughout reoxygenation (R). **D** Stacked bar graphs representing percent red puncta throughout R. mCherry puncta: *n* = 4 biological replicates, 44,536 objects. Scale bar = 10 µm.

Our lab has recently developed an unbiased machine learning mitochondrial morphology classification system to accurately quantify mitochondrial morphology in primary cortical neurons under control conditions and subjected to different stressors^43^. The classification system was based on four distinct mitochondrial morphologies: networks, unbranched, swollen, and punctate. Because mitochondrial dysfunction, characterized by mitochondrial fragmentation and mitochondrial swelling, is well accepted as the driving force of neuronal death following IR injury^8,9,18,44–46^, we investigated whether swollen mitochondria were preferentially cleared following OGD/R. Therefore, we developed a model to classify distinct mitochondrial morphologies in our MitoQC mice (Fig. 3A, Supplementary Material, and Supplementary Fig. 1).

**Fig. 4:**
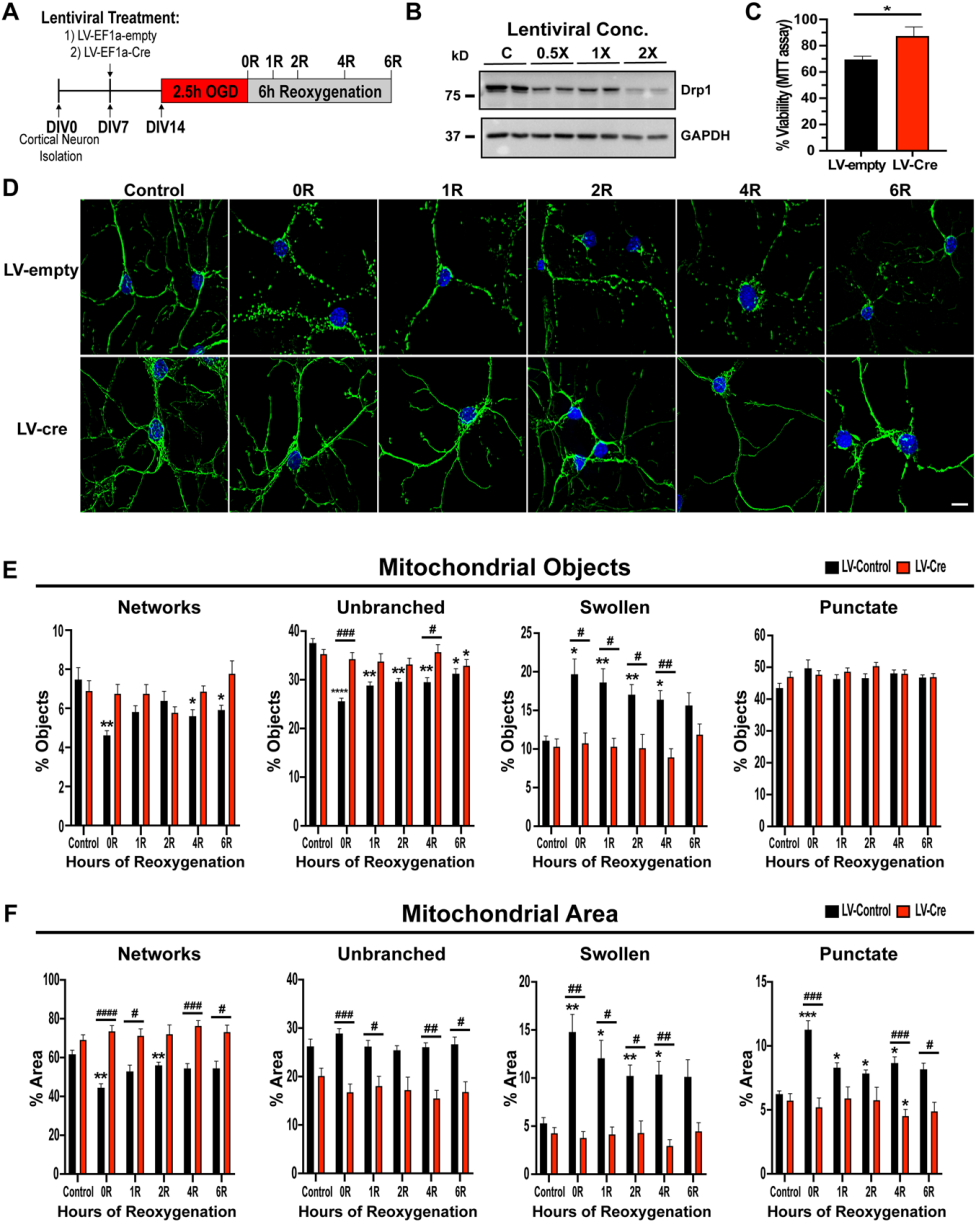
Drp1 KO stabilizes mitochondrial morphology during OGD/R. **A** Schematic diagram of experimental design. **B** Western blot of Drp1 knockout after Lentiviral transduction in *Drp1*^*fl/fl*^ cortical neurons. **C** Viability of *Drp1*^*fl/fl*^ cortical neurons after 2.5 h OGD and 6 h reoxygenation (R). Comparison was made by Student’s *t*-test, *n* = 10. **D** Representative images of *Drp1*^*fl/fl*^/*QC*^*Ki/+*^ primary cortical neurons (GFP channel), infected with either LV-EF1a-empty or LV-EF1a-cre, and subjected to OGD/R. **E** Individual comparisons of percent mitochondrial objects for each morphology over OGD/R. **F** Individual comparisons of percent mitochondrial area for each morphology over OGD/R. Two-way ANOVA was used to detect differences across time and between conditions. Multiple comparisons across time were assessed by comparing the means of each time point with the mean of the control and were calculated using Dunnett’s post-hoc analysis. Multiple comparisons between conditions were computed using Sidak’s post-hoc analysis. *p < 0.05; **p < 0.01; ***p < 0.001; ****p < 0.0001 across time, ^#^*p* < 0.05; ^##^*p* < 0.01; ^###^*p* < 0.001; ^####^*p* < 0.0001 between condition, *n* = 8 biological replicates per group. Scale bar = 10 µm.

To address this question, segmented mCherry puncta masks were generated from mitoQC images and analyzed using our classification system (Fig. 3B). Total mCherry puncta objects were counted from four biological replicates at each time point subjected to our standard OGD/R protocol (Fig. 1A), for a total of 44,536 individual objects, and groups were compared using a χ^2^ test (Fig. 4C). Of the 44,536 mCherry objects that were analyzed over all time points, the majority (89%) were classified as punctate (Fig. 3C, D). Unbranched morphology comprised 8.5% of mCherry objects, while swollen morphology comprised only 5.6% of mCherry objects. Not surprisingly, network morphology contained the lowest number of objects at 12, representing 0.02% of mCherry objects. Although the percentage of mCherry objects classified as a punctate morphology were not significantly different across time points (Fig. 4B), the total counts of punctate objects and total mCherry objects were increased at 4 and 6 h of reoxygenation (Fig. 3D), consistent with our previous results. For all classifications, mCherry objects demonstrated lower average areas when compared to mitochondrial objects (Supplementary Fig. 5).

Punctate mitochondria account for more than 40% of mitochondrial objects in primary neurons, while swollen mitochondria account for 10% (Fig. 4E). To ensure that the high percentage of mCherry punctate objects is not a result of the high number of punctate objects that exist within the cell, we normalized mCherry objects (mCherry-positive only) by the total mitochondrial objects (mCherry-positive + GFP-positive) (Supplementary Fig. 6). Our results show that 45–55% of punctate objects are recruited to lysosomes, while only 10–30% of swollen objects are mCherry-positive only. These data suggest that punctate mitochondria are the predominant morphology undergoing degradation.

### Drp1KO prevents mitochondrial fragmentation and cell death following OGD/R

Drp1 is widely accepted as the primary regulator of mitochondrial fission. Previous studies have demonstrated that, in both brain and heart, the pharmacological Drp1 inhibitor mdivi1 attenuates mitochondrial fragmentation and improves outcomes following in vivo and in vitro I/R injury^8,13,18,19^. However, these studies are limited by: (i) their qualitative/semi-quantitative measurement of mitochondrial dynamics; and (ii) the use of pharmacological agents that are recognized to have off-target effects^47^. Accordingly, we utilized our machine learning classification system as well as Lentiviral-Cre KO of *Drp1*^*fl/fl*^*/QC*^*Ki/+*^ primary cortical neurons to investigate the mechanistic role of *Drp1*KO in mitochondrial dynamics and cell death during OGD/R (Fig. 4). For all experiments, 2× lentiviral concentration was utilized (based on preliminary optimization studies, Supplementary Fig. 7), and neurons were subjected to OGD at 7 days post transduction (Fig. 4A, B). Under these conditions, neurons demonstrated a 75% loss of Drp1 protein (Fig. 4B and Supplementary Fig. 7).

We quantified mitochondrial objects and the area of mitochondria in each morphologic state between conditions LV-Ef1a-Cre (i.e., Drp1 KO) and LV-EF1a-empty (negative lentiviral control). Morphological classification in lentiviral-control neurons revealed significant reductions in networks and unbranched mitochondrial objects in cells subjected to OGD/R vs control (Fig. 4E). *Drp1*KO mitigated the OGD/R-associated reduction in unbranched mitochondria (Fig. 4E), and a similar (albeit non-significant) group trend was observed for network objects (*F* = 4.526, *p* = 0.0516; Fig. 4E). In agreement with the mitochondrial object counts, the area of mitochondrial networks as a percent of total mitochondrial mass was significantly reduced at early reoxygenation time points (Fig. 4F). Moreover, this reduction in mitochondrial area was attenuated in *Drp1*KO neurons, thereby implicating Drp1 in mitochondrial network fragmentation during OGD/R (Fig. 4F). Although the number of unbranched mitochondria decreased during OGD/R, the area of unbranched mitochondria was not reduced following reoxygenation. *Drp1*KO inhibited the decrease in number of unbranched mitochondrial objects, and the area occupied by unbranched objects remained comparable in cells subjected to OGD/R vs control (Fig. 4E, F). This suggests that unbranched mitochondria remain a stable percentage of the total mitochondrial pool but increase in size to compensate for the decrease in object number. Interestingly, mitochondrial area in the unbranched state was significantly lower in *Drp1*KO neurons compared to lentiviral control. These data suggest that Drp1 is essential for maintaining unbranched mitochondrial size, and *Drp1*KO results in shorter unbranched mitochondria without disrupting the number of mitochondria under control conditions (Fig. 4F).

Next, we investigated the effect of *Drp1*KO on the total number and area occupied by punctate and swollen mitochondrial morphologies. Interestingly, there was no increase in the number of punctate objects in lentiviral-control neurons in response to OGD/R; however, there was a significant increase in the total area of mitochondria in a punctate state—an increase that was attenuated with *Drp1*KO (Fig. 4E, F). These data suggest that punctate mitochondria are larger during OGD/R, and that Drp1 activity may induce a rapid transition from punctate mitochondria to a swollen mitochondrial phenotype. Most notably, both the number and the total area of mitochondria in a swollen state increased significantly in control neurons throughout OGD/R, and these increases were attenuated by *Drp1*KO.

Swelling of mitochondria is a well-documented harbinger of cell death in the setting of I/R^44–46^. Given our observation that *Drp1*KO reduced mitochondrial fragmentation and transition to a swollen phenotype, we investigated the effect of *Drp1*KO on neuronal viability following 2.5 h OGD and 6 h reoxygenation. As expected^8,13,18,19^, *Drp1*KO resulted in a 20% improvement in viability (Fig. 4C), suggesting that Drp1 contributes to neuronal death during I/R.

### Mitophagic flux is independent of mitochondrial fission

**Fig. 5:**
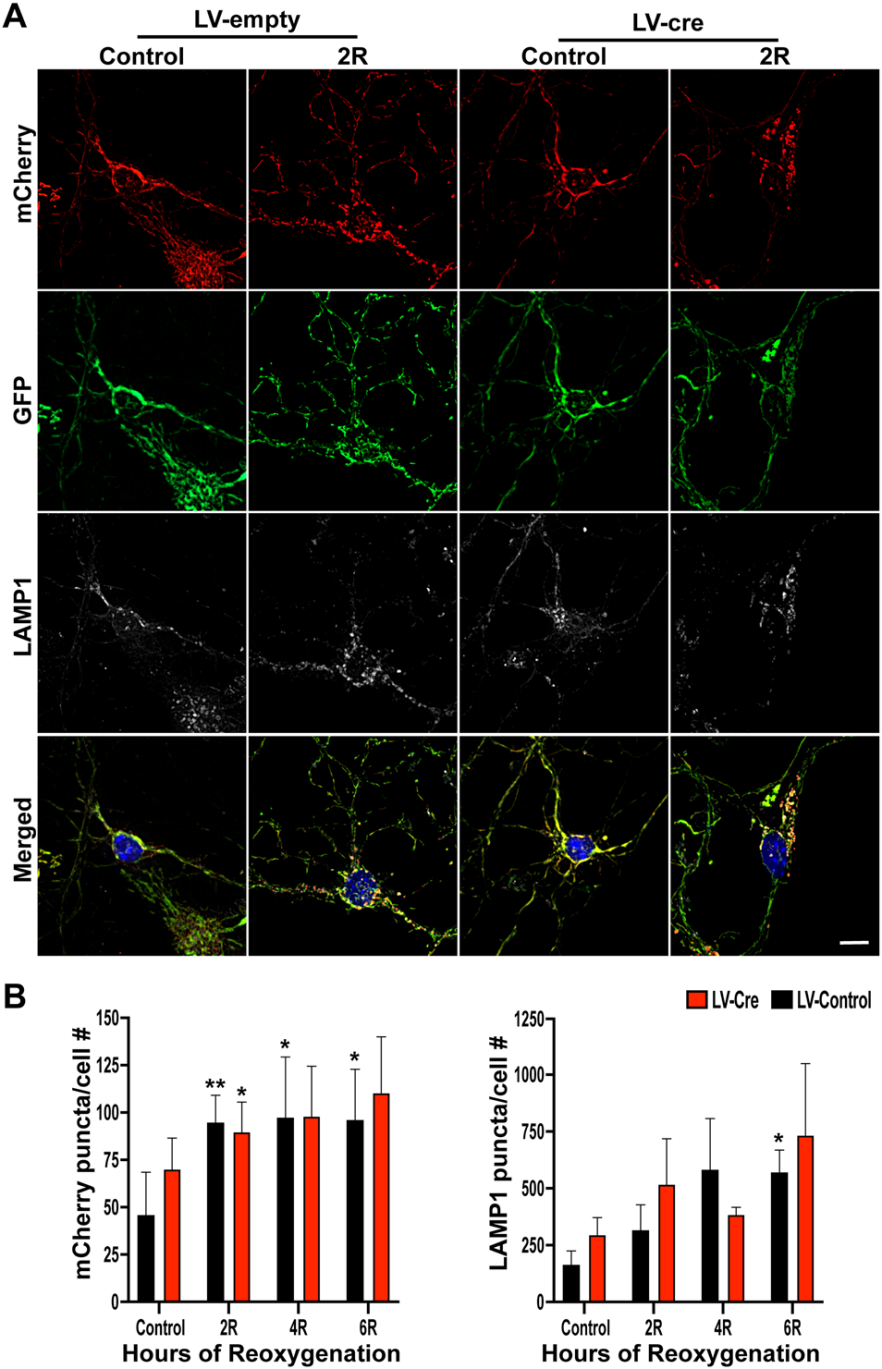
Mitophagic flux is independent of Drp1. **A** Representative images of *Drp1*^*fl/fl*^/*QC*^*Ki/+*^ primary cortical neurons, infected with either LV-EF1a-empty or LV-EF1a-cre, subjected to OGD/R and analyzed for the presence of mCherry puncta. **B** Quantification of mCherry puncta per cell and total LAMP1 puncta per cell during OGD/R. Two-way ANOVA was used to detect differences across time and between conditions. Multiple comparisons across time were assessed by comparing the means of each time point with the mean of the control and were calculated using Dunnett’s post-hoc analysis. Multiple comparisons between conditions were computed using Sidak’s post-hoc analysis. *p < 0.05; **p < 0.01 across time, ^#^*p* < 0.05 between condition, *n* = 8 biological replicates per group. Scale bar = 10 µm.

The neuroprotective effects of Drp1 inhibition and knockdown seen in our study and by others has been attributed to reduction in mitochondrial fragmentation^5–8,13,18,19^. However, there is an emerging paradigm implicating an expanded, mechanistic role of Drp1 beyond mitochondrial fission: Drp1 may participate in regulating the segregation of dysfunctional mitochondria tagged for degradation^10,20,21^. Although inhibition of fission during I/R may improve outcomes, it remains unclear whether this presumably favorable effect is accompanied by a reduction in the clearance of dysfunctional mitochondria accumulated during reperfusion. Therefore, we next investigated the effects of *Drp1*KO on mitophagic flux during OGD/R by infecting *Drp1*^*fl/fl*^*/QC*^*Ki/+*^ neurons with LV-EF1a-empty or LV-EF1a-Cre. Neurons were analyzed for the accumulation of mCherry puncta as an indicator of mitophagic flux (Fig. 5A). mCherry accumulation was significantly increased in control neurons throughout reoxygenation, indicating an increase in mitophagic flux in response to OGD/R (Fig. 5B)—an effect that was not altered in *Drp1*KO neurons (Fig. 5B). Similarly, neurons subjected to OGD/R displayed an increase in total LAMP1 particles (*p* < 0.05 at 6 h post R) with no difference between control and *Drp1*KO groups (Fig. 5B). These data suggest that induction of mitophagic flux following OGD/R is independent of Drp1.

## Discussion

The mitochondrial network is maintained in a healthy state through the fission and degradation of damaged or dysfunctional mitochondria. However, the specific role and integration of mitochondrial dynamics and mitophagy mechanisms^10,15,21^ in the progression of I/R injury remains unclear. Indeed, mitochondrial fission is necessary for mitophagy by producing mitochondria that meet the size requirement for engulfment by autophagosomes^48^. Furthermore, evidence suggests that Drp1, the primary mediator of fission, contributes to selection and tagging of dysfunctional mitochondria for degradation, and thus constitutes a molecular link between fission and mitophagy^10,20,21^. In this study, we used a novel approach to demonstrate activation of fission and mitophagy following OGD/R and interrogated the role of Drp1 in the process of fission, mitophagy, and cell death. We report that, during OGD/R, mitochondria are targeted for mitophagy by PINK/Parkin and that mitochondria are transported to lysosomes through the endosomal pathway. Our results suggest that, in addition to the well-documented increase in mitochondrial fragmentation, mitochondrial swelling is a rapid and sustained consequence of OGD/R. However, although mitochondrial swelling is associated with damaged mitochondria, ROS production, and apoptosis, we found that the majority of mitochondria that undergo mitophagy have a punctate morphology. Lastly, we make the novel observation that, while OGD-induced fission and transition to a swollen morphology are Drp1-dependent processes, mitochondrial clearance through mitophagy is independent of Drp1.

Mitochondria are continuously restructured and recycled in response to cellular demands and to maintain the health of the mitochondrial network; mitochondrial dynamics regulates mitochondrial structure through fusion and fission, while mitophagy controls the degradation of mitochondria through selective autophagy^49^. Imbalance or dysfunction of mitochondrial maintenance is associated with development of neurological diseases, including neurodegenerative conditions and acute neurological injury^26^. However, despite a likely role of mitochondrial dynamics and mitophagy in the development of I/R injury, the causal mechanisms are less clear.

### Mitophagy, fission, and cerebral I/R injury

Recent studies report a robust increase in mitophagy following cerebral I/R^12,50,51^ and OGD in neurons^52^. In accordance with the concept that mitophagy eliminates damaged mitochondria, upregulation of mitophagy purportedly provides a beneficial effect against I/R injury^13,14,53^. Consistent with the observed increase in mitophagy during I/R injury, there is a consensus that fission is induced following OGD^9,54^ and transient ischemia in vivo^55,56^. Although fission is essential for isolating damaged mitochondria, evidence suggests that upregulation of fission contributes to the development of neurological I/R injury^18,57^. In apparent contrast to these paradigms, additional studies have found that fission has been linked to neuroprotection^13,15^, while excessive mitophagy may exacerbate injury^17^—conflicting observations that have led investigators to propose that a balance between mitophagy and fission is key in mitochondrial maintenance and cell survival^17^.

Consistent with the evidence in the field, we demonstrate that mitophagy and fission are increased in neurons during reoxygenation. As expected, punctate mitochondria were the preferred morphology for mitophagy. Previous studies visualizing mitochondria inside autophagosomes by confocal and electron microscopy demonstrated that these sequestered mitochondria are generally small (<1 μm in diameter)^58–62^. Interestingly, few swollen mitochondria (5.6%) underwent mitophagy, which may have contributed to their accumulation during reoxygenation. It is unclear why swollen mitochondria evade elimination; however, their larger size relative to punctate mitochondria may limit their inclusion in autophagosomes. Another possible explanation is that the increase in mitochondrial membrane permeability, the underlying cause of swelling, may disrupt identification by the PINK/Parkin pathway and endosomal sorting complexes required for transport.

The MitoQC dual-fluorophore that provides the foundation for our analysis is designed to allow identification of mitochondria inside lysosomes by neutralizing pH-sensitive GFP. A potential limitation of this technique is that it is difficult to distinguish with certainty whether the mCherry puncta within lysosomes are, as we propose, mitochondria with mCherry-only fluorescence, or whether the lysosomes are filled with mCherry that has been freed from degraded mitochondria. However, our estimations of the size of punctate mitochondria as determined by mCherry fluorescence are comparable to those reported in previous studies^58–61^. Moreover, lysosomes are ~1.2 μm in diameter^62^, suggesting that mCherry objects present in lysosomes represent individual mitochondria.

### Molecular role(s) of Drp1

The large dynamin-related GTPase, Drp1, is the primary regulator of fission and has been implicated as a driver of I/R-induced fission and cell death^56,63,64^. However, recent studies have challenged the causal role of fission and Drp1 in cell death^47^. Findings in support of this paradigm have often utilized mdivi1, a chemical inhibitor of Drp1 translocation, and interpretation of these data may be confounded by evidence that mdivi1 is not selective for Drp1. Rather, mdivi1 purportedly acts as an inhibitor of NADH dehydrogenase and indirectly suppresses fission by inhibiting complex I-mediated ROS generation^47^.

Our current study, using a genetic (rather than pharmacologic) strategy to modify Drp1, provides robust mechanistic support for the concept that increased fission and cell death following OGD is, indeed, Drp1 dependent. Furthermore, we report that Drp1 expression plays a causal role in mitochondrial swelling observed during reoxygenation—results that are consistent with those of a previous study, showing that Drp1 KO in muscle cells caused a swollen mitochondrial phenotype^65^. This may be explained by a possible role of Drp1 in mitochondrial outer membrane permeabilization through the association and recruitment of pro-apoptotic proteins such as Bax and Bak—a process that triggers mitochondrial swelling, the release of cytochrome *c*, and cell death^5,66–69^.

Interestingly, in contrast to previous studies, *Drp1* KO did not alter the morphology of the mitochondrial network under baseline conditions (i.e., in neurons not subjected to OGD/R). These data suggest that, under normoxic conditions, the mitochondrial network at 7 days following conditional KO of *Drp1* is in a state of equilibrium, and the consequences of *Drp1* KO only manifest after exposure to the stress of OGD/R.

### Relationship between fission and mitophagy

The systems that regulate mitochondrial maintenance are likely integrated. On a physical level, only fragmented mitochondria will fit inside autophagosomes^70^, while fusion and elongation rescues mitochondria from mitophagy^48^. Moreover, on a molecular level, fission is associated with mitochondrial depolarization^10^ (a targeting signal for mitophagy through the PINK/Parkin pathway^10,48^), while Parkin rapidly ubiquitinates Mitofusins 1 and 2, preventing fusion and thus promoting mitophagy.

Despite evidence that fission regulates mitophagy^10,15^, the concept that Drp1 plays a direct mechanistic role in regulating mitophagy is controversial^71^. Evidence obtained in yeast^72^ and mammalian cells^10^ suggest that Drp1 (and Dnm1, the yeast homolog) is required for mitophagy while, in contrast, others have reported that *Drp1* KO had no effect on mitophagy^73^ or hypoxia-induced upregulation of mitophagy^74^, but may protect mitochondria from PINK/Parkin-mediated “wholesale mitophagy”^11^. Our results, showing no effect of *Drp1* KO on induction of mitophagy or mitophagic flux, are consistent with these latter observations and argue against a direct role of Drp1 as a molecular mediator of mitophagy in our neuronal cell model.

### Summary

In this study, we provide evidence that mitochondrial fragmentation, mitochondrial swelling, and mitophagy are upregulated in primary neuronal cells subjected to OGD/R. While fragmentation and swelling are attenuated by *Drp1* KO, we make the novel observation that changes in mitophagy are independent of Drp1. Our results are consistent with the paradigm that dysregulation of mitochondrial maintenance drives neurological I/R injury and suggests that mitophagy serves as a protective mechanism to eliminate dysfunctional mitochondria in this disease process.

## Methods

### Animals

All experimental procedures were performed in accordance with institutional guidelines and approved by the Institutional Animal Care and Use Committee under protocol #IACUC-18-08-0767 at Wayne State University and under protocol #PRO00009531 at the University of Michigan. MitoQC reporter mice (C57BL/6-Gt(ROSA)26Sortm1(CAG-mCherry/GFP)Ganl/J) were provided by Dr Ian Ganley, University of Dundee, Scotland, UK^22^. *Drp1* floxed (*Drp1*^*fl/fl*^) mice (*Dnm1l*^*tm1.1Hise*^) were provided by Dr Hiromi Sesaki, Johns Hopkins, Baltimore, MD^75^. WT mice (B57BL/6Crl) were purchased from Charles River Laboratories.

### Primary cortical neuron isolation and culture

Brains were isolated on postnatal day 0–2 (P0–P2), and cortical neurons were seeded separately for individually biological replicates. Tissue was digested with enzyme digestion solution (1× Hibernate complete medium, 0.06 mg/ml L-cysteine (Sigma, 778672), 1.4 × 10^−4^ N NaOH (Sigma, 43617), 10 ng/ml APV (2-Amino-5-phosphonopentanoic acid, Sigma, A-5282), 50 µl of Papain (Worthington, LS 03126)) and incubated at 37 °C for 30 min. After 30 min, the tissue was washed with Dulbecco’s phosphate buffered saline twice and replaced with Hibernate complete medium, and the tissue was dissociated by pipetting up and down 25 times to generate a cell suspension. The suspension was passed through a 40-micron filter to separate tissue debris. Cells were seeded at 190,000 cells/cm^2^ and incubated for 30 min at 37 °C in 5% CO_2_. After 30 min, media was replaced with neurobasal complete medium (1× Neurobasal Plus medium (Gibco, A3582901), 1% B27 plus (Gibco, A3653401), 0.5 mM Glutamax Supplement (Gibco, 35050061), and 1% Penn/Strep (Gibco, SV30010)) and cultured at 37 °C in 5% CO_2_. Half-media changes occurred every 3–4 days with neurobasal complete medium. Cultures were randomly assigned for lentiviral treatment as well as different time points of OGD/R.

### Oxygen-glucose deprivation and reoxygenation (OGD/R)

OGD was achieved utilizing an O_2_ Control InVitro Glove Box (Coy Lab Products). The hypoxic chamber was maintained at 0.1% O_2_ and 5% CO_2_. OGD media, composed of 0.20 g/l CaCl_2_ (Spectrum, CA138500GM), 0.4 g/l KCl (Fisher Chemical, P217-500), 0.097 g/l MgSO_4_ (Fisher Chemical, M65-500), 6.8 g/l NaCl (Fisher Chemical, S2711), 2.2 NaHCO_3_ (Acros Organics, AC447102500), 0.14 g/l NaH_2_PO_4_-H_2_O (Fisher Chemical, S369-500, and 0.01 g/l Phenol red (Fisher Chemical, P74-10), was bubbled with 95% N_2_/5% CO_2_ inside of the of the hypoxic chamber for 60 min. Cells were transferred into the hypoxic chamber, washed 2× with OGD media, and then incubated with OGD media inside the hypoxic chamber for 150 min. After OGD, cells were removed from the hypoxic chamber, media was replaced with Neurobasal medium without antioxidants (complete neurobasal medium with B27-AO (Gibco, 10889038)), and incubated at 37 °C in 5% CO_2_ for 6 h.

### Lentiviral transduction

Cells were infected with lentivirus on DIV 7. Experiments were performed 7 days post transduction (DIV 14). Lentiviral plasmids and lentivirus were generated by the vector core at the University of Michigan (Thomas Lanigan, PhD, Director): Lenti-EF1a-Cre-VSVG, Lenti-EF1a-VSVG, and Lenti-EF1a-GFP-VSVG. The EF1-alpha promoter, possessing a broad host range, demonstrated robust, constitutive, and long-term expression of both Cre-recombinase and GFP in our primary cortical neuron cultures. Vesicular stomatitis Indiana virus G-protein (VSVG) envelope was used to enhance viral entry. Viruses were made in a concentrated 10× form in Neurobasal medium. Titration curves were performed with Lenti-EF1a-VSVG and Lenti-EF1a-GFP-VSVG to evaluate toxicity and transduction efficiency, respectively. Toxicity was tested at 0.5×, 1×, 2×, 4×, and 5× concentrations. Transduction efficiency was tested at 0.5× and 1× concentrations.

### MTT assay

Viability was assessed after 2.5 h of OGD followed by 6 h of reoxygenation using Thiazolyl Blue Tetrazolium Bromide (MTT) assay. Following the 2 h incubation with MTT, media was removed and replaced with MTT solubilization solution (10% Triton-X100 and 12 mM hydrochloric acid (Fisher Chemical, A144SI-212) in isopropanol (Fisher Chemical, A419-4)) and placed on a shaker at room temperature for 15 min. Then, 100 µl of each sample was then pipetted into a 96-well plate and analyzed for absorbance at 570 nm utilizing a PerkinElmer Multimode Plate Reader containing Enspire interface software.

### Mitochondrial isolation

To obtain mitochondria, primary cortical neurons were collected in homogenization-A buffer (10 mM HEPES (pH7.5) (Sigma, H4034), 1 mM EDTA (Sigma, E6758), 1 mM EGTA (Sigma, 03777), 100 mM KCl (Sigma, P9333), 210 mM mannitol (Sigma, M4125), 70 mM sucrose (Sigma, S0389), and 1× Halt^TM^ protease and phosphatase inhibitor cocktail (ThermoFisher, 78440)) and homogenized utilizing a Teflon Potter-Elvehjem homogenizer (10 strokes at 300 rpm) (Thomas Scientific, 3432S90). Cell homogenates were centrifuged at 1000 × g for 10 min at 4 °C. The supernatant (containing mitochondria and cytosol) was centrifuged at 10,000 × g for 10 min at 4 °C. The supernatant was collected as the cytosolic fraction. Mitochondrial pellets were washed once and resuspended with fresh homogenization-A buffer and sonicated. Whole cell pellets were saved and sonicated to be used as whole cell lysate samples.

### Western blot antibodies

Dynamin-like protein 1 (Dlp1/Drp1) (Clone 8) mouse antibody (BD Transduction Laboratories, BD611112, 1:1,000, 2% milk), mono- and polyubiquitinylated conjugated mouse monoclonal antibody clone FK2 (Enzo, BML-PW8810, 1:1,000, 5% milk), PINK1 rabbit polyclonal antibody (Novus Biologicals, BC100-494, 1:1,000, 2% BSA), Parkin (Prk8) mouse monoclonal antibody (Cell Signaling, 4211, 1:1,000, 2% milk), Rab5 (C8B1) rabbit monoclonal antibody (Cell Signaling, 3547, 1:1,000, 5% BSA), VDAC (D73D12) rabbit monoclonal antibody (Cell Signaling, 4661, 1:1,000, 2% BSA), GAPDH (14C10) rabbit monoclonal antibody (Cell Signaling, 2118, 1:10,000, 2% BSA), and LC3 A/B rabbit antibody (Cell Signaling, 4108, 1:1,000, 2% BSA).

### Western blot

Cells were scraped in homogenization-A buffer (with MG132 proteasome inhibitor when probing for ubiquitination), sonicated, and the protein concentration was measured using the Bradford Plus Assay Reagent (ThermoFisher, PI23236). Polyacrylamide gels (10% 29:1 polyacrylamide/bisacrylamide (Fisher BioReagents, BP1408-1), 375 mM Tris pH 8.8 (Fisher BioReagents, BP152-1), 0.1% sodium dodecyl sulfate (Sigma, L3771), 0.1% ammonium persulfate (APS, Sigma, #A3678), and 0.1% TEMED (GE Healthcare, #45-000-226)) were loaded with 10 μg of whole cell lysates and 5 μg of mitochondrial samples and transferred to nitrocellulose membranes. Membranes were incubated with indicated primary antibodies at 4 °C overnight. Membranes were then washed with Tris-buffered saline and 0.1% Tween (TBST, Fisher Scientific, BP337500) and incubated in secondary antibodies for 60 min at room temperature. Membranes were washed 3× with TBST, for 5 min. The membranes were then incubated in SuperSignal West Pico Plus Chemiluminescent Substrate (ThermoFisher, 34577), imaged utilizing a BioRad ChemiDoc XRS+ imager and quantified by densitometry using ImageJ software.

### Immunofluorescence antibodies

MAP2 chicken polyclonal antibody (Abcam, ab5392, 1:1,000), Parkin rabbit polyclonal antibody (ThermoFisher, PA5-13399, 1:50), PINK1 rabbit polyclonal antibody (Novus Biologicals, BC100-494, 1:100), Lamp1 (1D4B) rat antibody (Santa Cruz Biotechnology, sc-19992, 1:100), and PINK1 rabbit polyclonal antibody (Novus Biologicals, BC100-494, 1:100). Secondary antibodies were donkey anti-mouse or donkey anti-rabbit HRP (Jackson Immuno Research Labs, NC9832458 and NC9736726), Alexa Fluor 488, Alexa Fluor 568, and Alexa Fluor 633 goat anti-rat (Life Technologies).

### Immunofluorescence

Proteins were visualized with immunofluorescence as previously described^9^. Briefly, cells were fixed with 3.7% PFA (ThermoFisher, 50980487) in 200 mM HEPES (Sigma, H4034) for 15 min at 37 °C. Coverslips were washed 3× with PBS and replaced with blocking solution (5% goat serum (Sigma, G9023) and 0.3% Triton-X100 (Acros Organics, 215682500) in PBS) for 60 min. Blocking solution was replaced with primary antibodies (with 1% BSA and 0.3% Triton-X100 in PBS) at 4 °C overnight. After primary incubation, coverslips were washed 3× in PBS and incubated with indicated secondary antibodies for 60 min at room temperature, shielded from light. Coverslips were subsequently washed and mounted using Fluoroshield with DAPI (Sigma, F6057).

### MitoQC analysis

For each coverslip containing MitoQC neurons, 12 random images were acquired utilizing a Zeiss Axio Observer.Z1 inverted microscope at 63× oil immersion. Each image was deconvolved using the regularized inverse filter method on Zeiss Zen Pro software. Images were further processed in Fiji ImageJ to segment and count mCherry-only puncta^76^. The following was performed using Fiji’s (ImageJ) batch processing feature in three separate phases: processing, segmentation, and red puncta analysis.

(1) Processing:

- Process > Subtract Background (Rolling Ball Radius = 10.0)
- Process > Filters > Unsharp Mask (Radius = 1.0 pixels, Mask Weight = 0.6)
- Process > Enhance Local Contrast (block size = 127, histogram bins = 256, maximum slope = 3.00, mask =;none, checkmark “fast” box)^77^
- Process > Filters > Median (radius = 2.0 pixels)

(2) Segmentation:

Trainable Weka Segmentation was employed to segment red particles. Trainable Weka Segmentation is a plugin in ImageJ that uses a combination of visualization tools and machine learning algorithms to produce a pixel-based segmentation^78^. The program was trained to segment “mitophagy” particles (red-only), “healthy mitochondria” (green/yellow), and “background” (“black”). Once the classifier was trained, photos were segmented using the category “probabilities” as an output.

(3) Red Puncta Analysis:

- Image > Stacks > Stacks to Image
- Close “Background” and “Healthy mitochondria” windows
- Select window titled “Mitophagy”
- Image > Adjust > Threshold (0.31, 1 × 10^31^)
- Process > Binary > Convert to Mask
- Analyze > Set Scale (9.7 pixels/micron
- Analyze > Analyze Particles (size = 0.0–Infinity, circularity = 0.0–1.0, show = nothing, check summarize)

Results of red puncta counts were tabulated into an excel sheet for eacg image and normalized to the number of cells analyzed.

### Machine learning mitochondrial classification

Machine learning-based classification of mitochondrial objects was performed as described in Supplementary Material and previous publication^43^. In brief, classification was performed in R computing language using the R Caret package^79^. Fiji ImageJ was used for image processing and mitochondrial object measurement collection^76^. R studio was used for all computation. The workflow was performed in two separate phases: Feature Extraction utilizing ImageJ and Random Forest Classification utilizing R studio.

(1) Feature Extraction:

The green channel of the MitoQC images was used for mitochondrial morphology analysis. Processing was done in the same workflow as above. Trainable WEKA segmentation was employed to segment green mitochondrial signal using “Labels” as an output. Segmented images were then analyzed to obtain measurements of each mitochondrial object. Workflow is listed below:

- Image > Type > 8-bit
- Image > Type > 16-bit
- Image > Type > 8-bit
- Process > Binary > Make Binary
- Analyze > Set Scale (9.7 pixels/micron)
- Analyze > Measure (if mean is ≤ 0.75 then invert (Edit > Invert), otherwise continue)
- BioVoxxel_Toolbox (ImageJ plugin) > Extended particle analyzer (Area = 0.3–Infinity, Extent = 0–1, Perimeter = 0–Infinity, Circularity = 0–1, Roundness = 0–1, Solidity = 0–1, Compactness = 0–1, AR = 0–Infinity, Feret AR = 0–Infinity, Ellipsoid angle = 0–180, Max Feret = 0–Infinity, Min Feret = 0–Infinity, Feret angle = 0–180, Coefficient of Variation = 0–1, Show = Nothing, Redirect to = none, Keep borders = none, check “display results”).

Measurements were then expanded using “extended descriptors” macro from the Applied Superconductivity Center^80^. A total of 32 measurements were recorded for each object: area, perimeter, circularity, feret, FeretAngle, Min Feret, aspect ratio (AR), Round, Solidity, Feret AR, Compactness, Extent, CircToEllipse Tilt, AR_Box, AR_Feret, Round_Feret, Compact_Feret, Elongation, Thinnes Ratio, Angle_0-90, Feret_Angle_0-90, Convexity, Roundness corrected to AR, area equivalent diameter, perimeter equivalent diameter, spherical equivalent diameter, interfacial density, Hexagonal Side, Hexagonal Perimeter, Hexagonal Shape Factor, Hexagonal Shape Factor Ratio, and Hexagonality.

(2) Random Forest Classifier:

Hand-classified mitochondrial objects (*n* = 2342) containing the 32 measurements (predictors) listed above were imported into R Studio as the train/test set. These objects were labeled with one of four different classifications: networks (*n* = 236), unbranched (*n* = 851), swollen (*n* = 458), or punctate (*n* = 797). The data were then split based on their classifications into the training (80%) and the test (20%) sets using the createDataPatition function from the caret package^79^. A random forest algorithm (rf) from the caret package was trained using the training set through 25 iterations utilizing 32 predictors. The final model consisted of 500 trees with an mtry = 2, which refers to the number of randomly selected predictors considered at each split within the tree.

### Statistical analysis

Statistical analysis was performed using GraphPad Prism. Principal component analysis was done in R using the built-in function prcomp(). Data are presented as means ± SEM. All experiments were independently repeated and data were collected from four to eight biological replicates with two to three technical replicates for each experiment. Based on variability, power analysis was conducted to determine appropriate sample size. Differences between three or more groups were analyzed using one-way ANOVA with either Tukey post-hoc test (for comparisons among all groups) or Dunnett’s post-hoc test (for comparisons to control). Student’s *t*-test was used to detect differences between two groups. Differences between conditions across time (Lenti-cre vs Lenti-empty) were analyzed using a two-way ANOVA with Dunnett’s post-hoc analysis for differences across time points compared to control. Multiple comparisons to detect differences between conditions were computed using Sidak’s post-hoc test. Differences were considered significant when *p* < 0.05.

## Supplementary information

Supplementary Material

## Data Availability

R functions written for machine learning training and morphological prediction are available in our GitHub repository (https://github.com/sanderson-lab/mitomorphology)^79^.
