# Supplementary material for: Mitochondrial fission and mitophagy are independent mechanisms regulating ischemia/reperfusion injury in primary neurons
Source: Cell Death Dis. 2021 May 12;12(5):475. doi: 10.1038/s41419-021-03752-2 (PMC8115279; doi:10.1038/s41419-021-03752-2)
Supplement: Supplementary file 1 — Supplementary Material [file 41419_2021_3752_MOESM1_ESM.pdf]

Supplemental Material.

Mitochondrial classification - We first identified 4 distinct mitochondrial morphologies: networks, unbranched, swollen, and punctate (Supplemental Fig. 1A). To validate the independence of these observed morphologies, mitochondrial objects (mitochondrial localized-GFP) were segmented and measured using 32 standard indices of size and shape descriptors (Supplemental Fig. 1B-C). Comparison of morphologies across size and shape descriptors identified distinct groupings of characteristics in these morphologic categories. Network objects were generally large in area and had low values for circularity. Unbranched objects generally had high aspect ratio (AR) values and low values for circularity. In contrast swollen and punctate objects contained high values for circularity with low values in AR, but differed in area and interfacial density. Swollen objects were larger in area, but had smaller values for interfacial density when compared to punctate mitochondria. Principal component analysis further revealed distinct groupings of our mitochondrial morphologies (Supplemental Fig. 1D). To summarize the differences of each morphology with respect to the 32 different indices, scaled measurements were computed, and variables were grouped by similarity (Supplemental Fig. 2).

To develop the model for classification, a total of 2342 mitochondrial objects were segmented from four different images (two from control conditions, and two from fragmented images), 32 measurements were collected for each object, and each object was classified by the operator based on visual inspection. The mitochondrial objects were split based on their classification into a training set (80%, 1874 objects) and a test set (20%, 469 objects) (Supplemental Fig. 3). A machine learning model was developed based upon the training set, and later validated using the test set. The model was built using Random Forest classification methodology and the final model consisted of 500 trees with an mtry = 2. Mtry refers to the number of variables randomly sampled as candidates at each split of the forest. The overall accuracy of the test set was calculated as 99.57% with a kappa of 99.39%, as calculated by a confusion matrix

(Supplemental Fig. 4). These data demonstrate that our machine-learning mitochondrial morphology classification system can be adapted to MitoQC primary cortical neurons and possesses high accuracy for the prediction of mitochondrial morphologies.

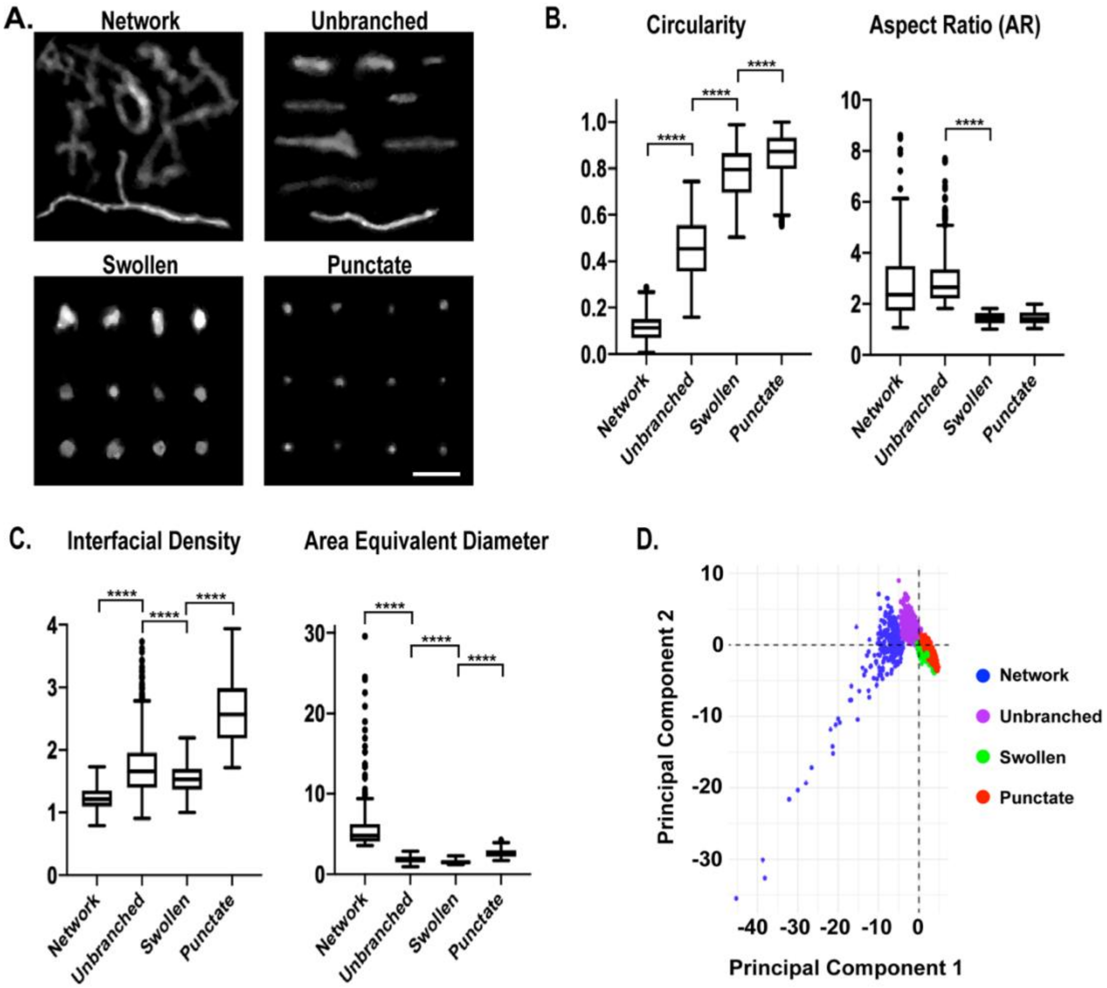

Supplemental Fig. 1: Identification of distinct mitochondrial morphologies. A) Four distinct morphologies were identified in primary cortical neurons from MitoQC transgenic mice: network, unbranched, swollen, and punctate. B-C) Mitochondrial morphologies differ significantly in circularity, aspect ratio, interfacial density, and area equivalent diameter. D) Principal component analysis of mitochondrial objects. Each point represents a single mitochondrial object, each color represents a different morphology: network (blue), unbranched (purple), swollen (green), and punctate (red). Differences across groups were determined using one-way ANOVA with Tukey post-hoc analysis. ****$p<0.0001$. Scale bar =

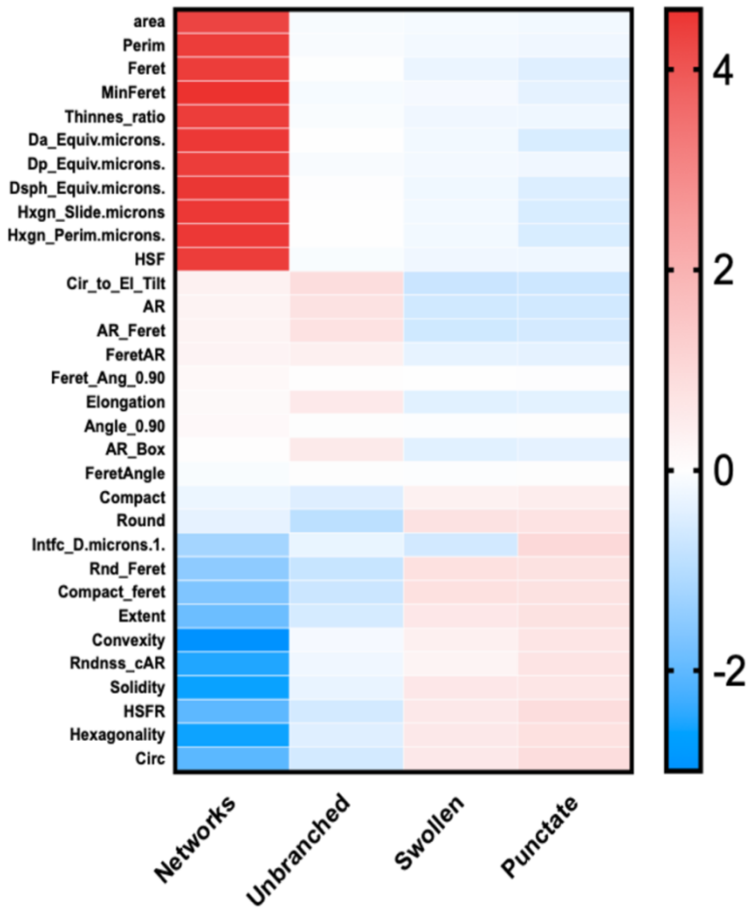

Supplemental Fig. 2: Comparison of scaled measurements across different morphologies. Thirty-two different measurements were collected for each mitochondrial object. Z-scores were calculated for each variable to compare differences across all morphologies. Variables were grouped based on similarity. Red color corresponds to a high z-

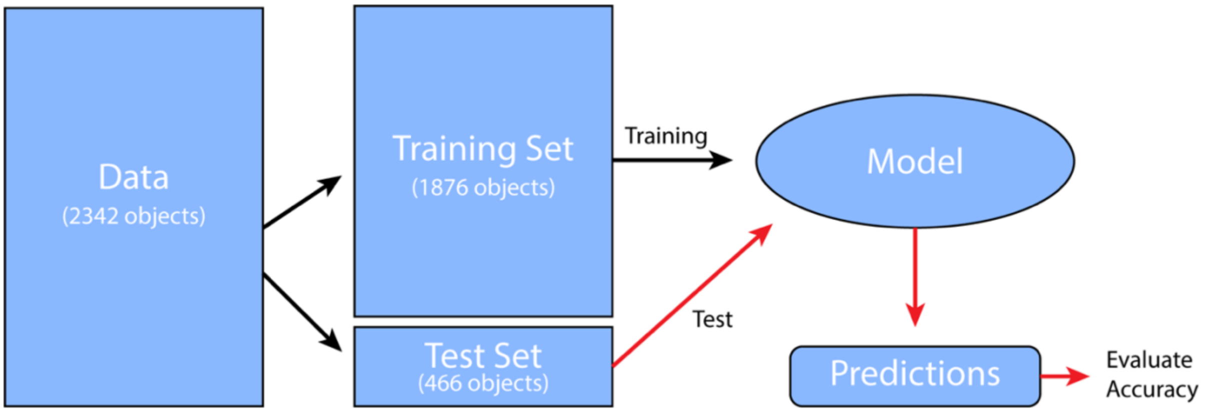

Supplemental Fig. 3: Classification model development. Mitochondrial objects (n=2,342) were classified by the operator based on visual inspection and split on the basis of their classification into a training set (80%) and a test set (20%). The training set was used to train the random forest algorithm over 25 different iterations. The test set was then later used to validate the accuracy of the model to correctly predict mitochondrial morphology.

A.

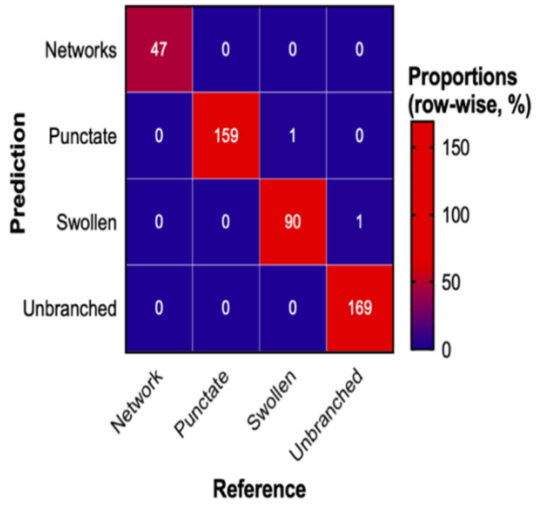

B.

Overall Accuracy: 0.9957 Kappa: 0.9939

	Network	Punctate	Swollen	Unbranched
Sensitivity	1.0000	1.0000	0.9973	0.9941
Specificity	1.0000	0.9968	0.9973	1.0000
Precision	1.0000	0.9938	0.9890	1.0000
Balanced Accuracy	1.0000	0.9984	0.9932	0.9971

Supplemental Fig. 4: Model performance. Model performance was analyzed using the test set data (468 objects). A) Confusion matrix shows that only two objects out of 468 were mislabeled. B) Overall accuracy was 99.57% with a kappa of 99.39%. Sensitivity, specificity, precision, and balanced accuracy for all morphologies were over 98.9%.

Area comparison between classified mCherry only objects vs. mitochondrial objects-

mCherry networks and mitochondrial networks had an average area of 8.822 $\pm$ 2.252 μm^2 versus 25.467 $\pm$ 56.991 μm^2, respectively. Unbranched mCherry objects and unbranched mitochondria had an average size of 1.440 $\pm$ 0.981 μm^2 versus 2.871 $\pm$ 1.812 μm^2, respectively. Swollen mCherry objects and swollen mitochondria had an average area of 1.681 $\pm$ 0.649 μm^2 versus 2.126 $\pm$ 1.066 μm^2, while punctate mCherry objects and punctate mitochondria had an average size of 0.382 $\pm$ 0.233 μm^2 versus 0.582 $\pm$ 0.215 μm^2. These data, summarized in Supplemental Fig. 5, suggest macro-mitophagy may occur on unbranched and network mitochondrial objects, but the objects are smaller than 10 μm^2.

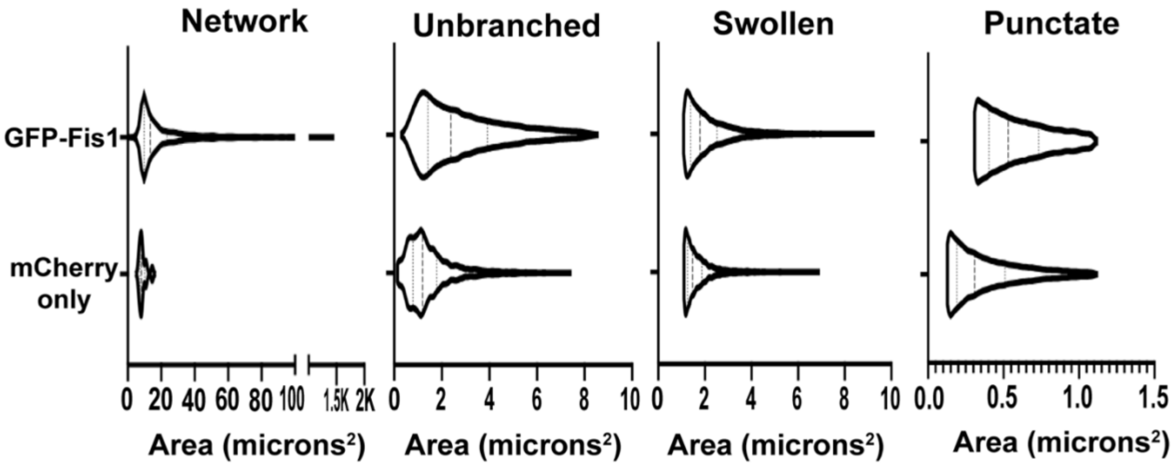

Supplemental Fig. 5: Comparison of classified object area between mCherry only puncta and GFP-fis1. Violin plots displaying relative distributions of area for mCherry puncta (mCherry only) and mitochondria (GFP-Fis1). mCherry puncta: n = 4 biological replicates, 44,536 objects. Mitochondria: n = 1 biological replicate, 43,434 objects.

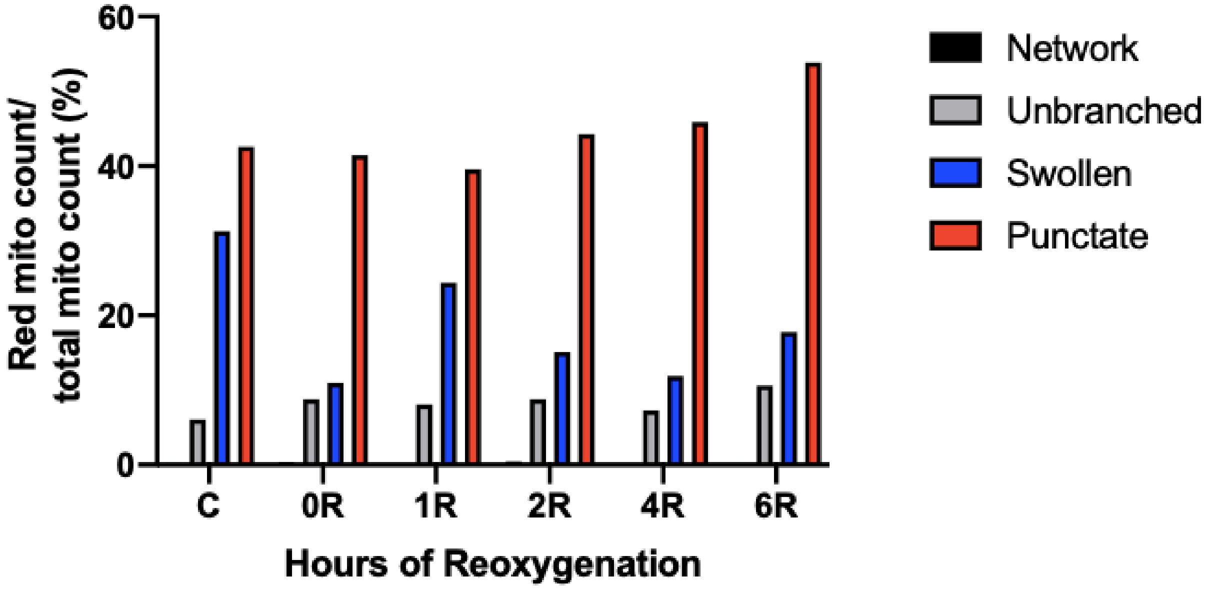

Supplemental Fig. 6: Number of mCherry objects normalized by total mitochondrial objects. Bar graph of the total mCherry (mCherry only) counts normalized by total mitochondrial counts (mCherry-positive + GFP-positive) for each classification. n = 4 biological replicates, 152,318 total objects.

Lentiviral Optimization-

To optimize target gene knockout with lentiviral-Cre delivery in primary cortical neurons we first conducted preliminary experiments to evaluate (i) cell viability, (ii) infection rate, and (iii) loss of protein target. Viral toxicity was determined with five escalating concentrations of lentivirus (LV-EF1a-empty-VSVG; concentrations 0.5X, 1X, 2X, 4X, 5X) and viability was assessed at 7 days post-transduction using the MTT assay. All concentrations had significant decreases in viability, with a ~25% reduction of viability at concentrations of 2X and above (Supplemental Fig. 6A). To validate neuron viral transduction efficiency, cortical neurons were transduced with 0.5X and 1X LV-EF1a-GFP-VSVG and co-labeled with immunofluorescence for the neuron marker microtubule-associated protein 2 (MAP2) on post-transduction day 3, 5, and 7 (Supplemental Fig. 6B). Infection rate was calculated as the number of MAP2-positive neurons containing GFP fluorescence divided by the total number of MAP2-positive neurons. By 7 days post-transduction the infection rate for both 0.5X and 1X LV-EF1a-GFP-VSVG was > 95% (Supplemental Fig. 6C). It was assumed that all concentrations greater than 1X, would yield similar results to 1X concentration in transduction efficiency. Knockout of Drp1 (Drp1KO) was evaluated by transducing *Drp1*$^{fl/fl}$ neuron cultures with 0.5X, 1X, and 2X LV-EF1a-cre-VSVG concentrations. Quantification of immunoblots revealed a 75% knockout of Drp1 protein in *Drp1*$^{fl/fl}$ neuron cultures with lentiviral-cre delivery (Supplemental Fig. 6D).

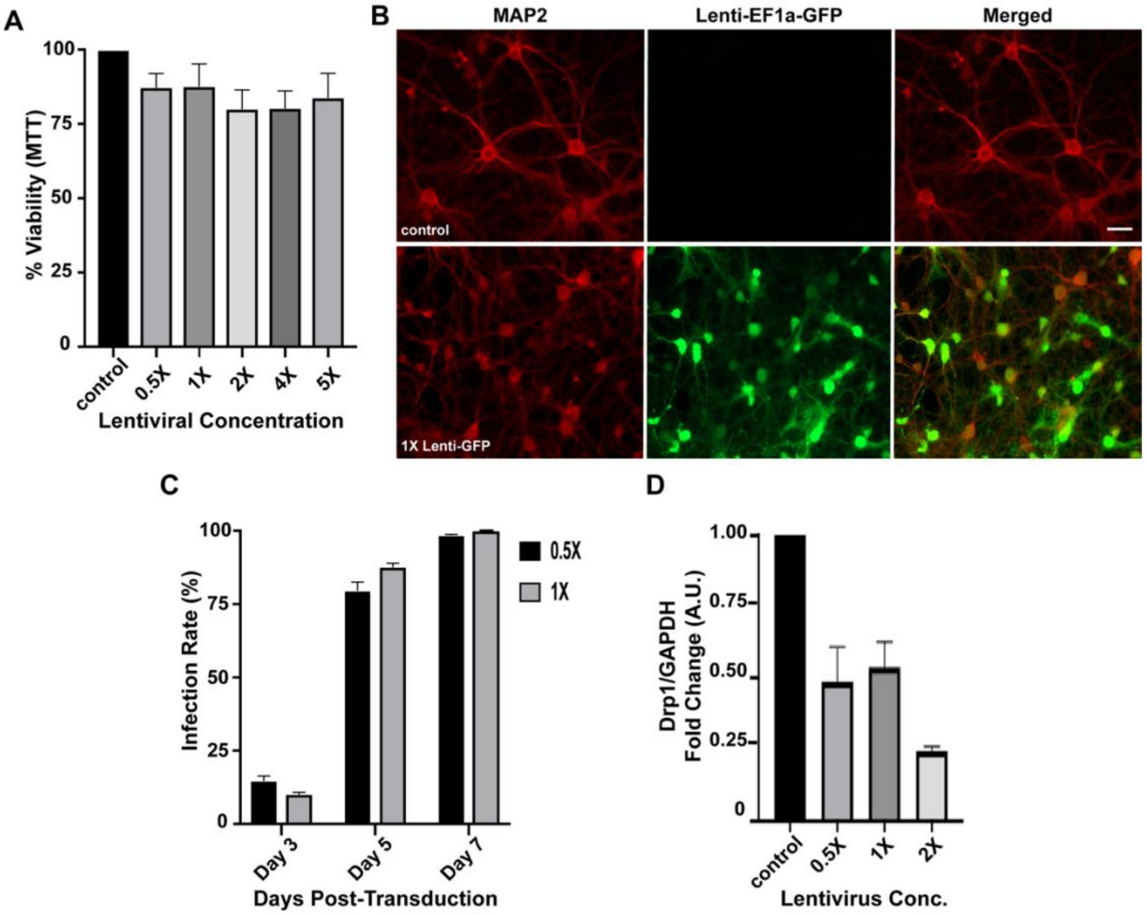

Supplemental Fig. 7. Lentiviral transduction of primary cortical neurons. A) Lentiviral toxicity was evaluated over 5 different concentrations of LV-EF1a-empty. Viability was assessed using the MTT assat, n = 4. B) Representative images of neuron viral transduction efficiency post-transduction day 7. Neuronal cultures were treated with 0.5X and 1X (shown) LV-EF1a-GFP and co-labeled with immunofluorescence for the neuron marker MAP2. C) Quantification of infection rate was calculated by the number of MAP-positive cells containing GFP divided by the total number of MAP2-positive cells, n = 3. D) Western Blot quantification of Drp1 knockout after transduction with 0.5X, 1X, and 2X LV-EF1a-cre in $Drp1^{fl/fl}$ primary cortical neurons, n = 3. Scale bar = 20 µm
